# Danger in Fruit Cans

**Published:** 1881-07

**Authors:** 


Danger in Fruit Cans.—The demand for cheap fruit cans has led to their manufacture from tin heavily alloyed with cheaper metals, such as lead, etc. The result is that such fruits as tomatoes, berries etc., containing large quantities of acid, when put in these cans becomes contaminated with the products of chemical reaction. This circumstance explains many of the incidents of poisoning from canned fruits, potted meats, etc., noticed almost daily in the newspapers. People will do well to pay a good price for their tin cans, thus securing a superior quality of ware. What would be still better would be to avoid the use of tin altogether, using nothing but glass.

